# High Throughput Field Phenotyping for Plant Height Using UAV-Based RGB Imagery in Wheat Breeding Lines: Feasibility and Validation

**DOI:** 10.3389/fpls.2021.591587

**Published:** 2021-02-16

**Authors:** Leonardo Volpato, Francisco Pinto, Lorena González-Pérez, Iyotirindranath Gilberto Thompson, Aluízio Borém, Matthew Reynolds, Bruno Gérard, Gemma Molero, Francelino Augusto Rodrigues

**Affiliations:** ^1^Department of Agronomy, Federal University of Viçosa, Viçosa, Brazil; ^2^International Maize and Wheat Improvement Center (CIMMYT), Texcoco, Mexico; ^3^KWS Momont Recherche, Mons-en-Pevele, France

**Keywords:** multi-temporal crop surface model, structure from motion, RGB camera, dense point cloud, drones, post-processed kinematic, wheat breeding, adjusted and predicted genotypic values

## Abstract

Plant height (PH) is an essential trait in the screening of most crops. While in crops such as wheat, medium stature helps reduce lodging, tall plants are preferred to increase total above-ground biomass. PH is an easy trait to measure manually, although it can be labor-intense depending on the number of plots. There is an increasing demand for alternative approaches to estimate PH in a higher throughput mode. Crop surface models (CSMs) derived from dense point clouds generated via aerial imagery could be used to estimate PH. This study evaluates PH estimation at different phenological stages using plot-level information from aerial imaging-derived 3D CSM in wheat inbred lines during two consecutive years. Multi-temporal and high spatial resolution images were collected by fixed-wing (*Plat*_*FW*_) and multi-rotor (*Plat*_*MR*_) unmanned aerial vehicle (UAV) platforms over two wheat populations (50 and 150 lines). The PH was measured and compared at four growth stages (GS) using ground-truth measurements (PHground) and UAV-based estimates (PHaerial). The CSMs generated from the aerial imagery were validated using ground control points (GCPs) as fixed reference targets at different heights. The results show that PH estimations using *Plat*_*FW*_ were consistent with those obtained from *Plat*_*MR*_, showing some slight differences due to image processing settings. The GCPs heights derived from CSM showed a high correlation and low error compared to their actual heights (*R*^2^ ≥ 0.90, *RMSE* ≤ 4 cm). The coefficient of determination (*R*^2^) between PHground and PHaerial at different GS ranged from 0.35 to 0.88, and the root mean square error (*RMSE*) from 0.39 to 4.02 cm for both platforms. In general, similar and higher heritability was obtained using PHaerial across different GS and years and ranged according to the variability, and environmental error of the PHground observed (0.06–0.97). Finally, we also observed high Spearman rank correlations (0.47–0.91) and *R*^2^ (0.63–0.95) of PHaerial adjusted and predicted values against PHground values. This study provides an example of the use of UAV-based high-resolution RGB imagery to obtain time-series estimates of PH, scalable to tens-of-thousands of plots, and thus suitable to be applied in plant wheat breeding trials.

## Introduction

Wheat (*Triticum* sp.) is among the leading food crops, and it is grown in a range of environments and geographical areas. It is highly relevant to the human diet, given its protein quantity, quality, and variety of derived products (Shewry et al., 2016). Lately, wheat has become the most important source of dietary protein and the second most important source of calories (carbohydrates) for humans (Shewry et al., 2016). During the 2017/2018 season, wheat was sown in 11.7% of the world’s arable land, corresponding to around 30% of world grain production (USDA, 2018). Wheat is vital for food security, supplying an affordable source of nutrition to a large portion of the global population, particularly millions of people with low-middle incomes, and is an essential crop for the composition of sustainable agricultural production systems (Walters et al., 2016; Hickey et al., 2019).

Wheat breeding for plant height (PH) has been historically used to reduce lodging and improve grain yield and quality (Reynolds et al., 2020). The identification and introduction of major dwarfing or semi-dwarfing genes were significant advancements in the wheat breeding work led by Norman Borlaug that enabled grain yield increase in most environments and contributed to the “Green Revolution” (Reynolds and Borlaug, 2006; Würschum et al., 2015). To date, PH continues to be one of the most critical and heritable traits in wheat breeding, also used to select suitable parental lines for hybrid breeding (Würschum et al., 2015; Barmeier et al., 2016). In addition, PH contributes to biomass production, as it is associated with increased photosynthesis due to a better light interception and distribution through the canopy in taller plants (Song et al., 2013).

PH is commonly calculated by measuring the distance between the upper boundary of the main photosynthetic tissues (excluding the awns) and the ground level using a simple metric ruler or a graduated stick (Torres and Pietragalla, 2012). Although simple, such assessment is costly, laborious, and prone to subjectivity, especially in extensive field trials (Holman et al., 2016; Hu et al., 2018). Manual ground measurements in the field are only feasible on a few plants per plot and can be biased by the lack of standardized criteria (Wilke et al., 2019). The temporal characterization of PH, that is, PH estimations at the various plant growth stages, could provide a better understanding of the mechanism of plant growth and its underlying genetic effects by providing insight into the environmental variables of this trait (Torres-Sánchez et al., 2013; Hassan et al., 2019a). Phenotyping under environmental field conditions is often considered a bottleneck in plant breeding programs (Yang et al., 2017; Rebetzke et al., 2019). Consequently, there is a need for faster, more accurate, and continuous measurements of PH.

HTP (high-throughput phenotyping) could be applied to accurately and efficiently conduct temporal PH characterization. The introduction of HTP approaches into breeding schemes can significantly improve the phenotyping standards for agronomic traits, contributing to better understanding of their genetic basis and diversity, as well as the environmental influences throughout the crop’s development cycle (Reynolds et al., 2020). Non-destructive data collected via ground-based and aerial HTP techniques are highly desirable for application in plant breeding since they can be used to assess different traits in large-scale field trials (Rodrigues et al., 2018; Loladze et al., 2019). In particular, aerial HTP platforms have become favored overground platforms, as they are suitable for use in large breeding trials and show a good trade-off between time, data accuracy, and resolution (Yang et al., 2017; Gracia-Romero et al., 2019; Zhao et al., 2019). The rapid development of sensors and unmanned aerial vehicles (UAVs), as well as that of image and data analyses algorithms and improved computer capacities observed in recent years, have enabled a broad range of possibilities for aerial HTP to measure plant traits such as PH, stand count, and vegetation indices, among others (Maes and Steppe, 2019; Matias et al., 2020; Morales et al., 2020). These aerial images are used as a proxy for the characterization of quantitative plant traits. Recent advances in remote sensing using UAV with sensors measuring the visible (RGB—red, green, and blue bands) and/or near-infrared (NIR) wavelengths have made it possible to create high-throughput, cost-effective, and accurate quantitative phenotyping datasets in wheat breeding programs (Singh et al., 2019; Reynolds et al., 2020).

Digital color images (i.e., RGB) collected from UAVs have been used for estimating PH in wheat through different settings and capabilities (Table 1). Feature matching and structure from motion (SfM) techniques applied to such imagery enable the generation of three-dimensional (3D) point clouds that can be used to reconstruct multi-temporal crop surface models (CSMs) from which PH can be estimated plot-wise (Singh et al., 2016; Hassan et al., 2019b). SfM photogrammetry is a method that uses a set of overlapped images to generate high-resolution topographic 3D-reconstructions. Through automatic extraction of corresponding feature points, this method optimizes the 3D location based on images taken from multiple perspectives, enabling a simple workflow (James and Robson, 2014; Nex and Remondino, 2014).

**TABLE 1:** Summary of published studies on the estimation of plant height (PH) for wheat from RGB imagery acquired using unmanned aerial vehicles (UAVs).

**References**	**GS**	**Platform—UAV**	**Camera/sensor**	**GSD cm/p**	**Total number of plots**	**Plot size (m)**	**Pixel extraction method**	***R*^2^ for PHground vs. PHaerial^‡^**	**RMSE for PHground vs. PHaerial (cm)^¶^**
Holman et al. (2016)	GS30 to GS61	Cinestar octocopter	Sony NEX 7 24.4 mgpx	1	300	9 × 3	99th percentile	0.52–0.99	1.5–9.9
Madec et al. (2017)	GS20 to GS69	Hexacopter	Sony ILCE-6,000 digital^$^	1	1,173	10 × 1.9	99.5th percentile	0.95–0.99	2.9–9.8
Hassan et al. (2019a)	GS41 and GS85	DJI inspires 1 model T600	Sequoia 4.0 16 mgpx	2.5	600	1.3 × 1.3	90th and 99th percentile	0.8–0.96	5.75
Li et al. (2019)	GS21 to GS87	DJI matrice 600 Pro	Zenmuse X5R RGB camera	0.5	170	3 × 0.23	Mean, median, 95th percentile, and standard deviation	–	–
Lu et al. (2019)	GS30 to GS69	DJI phantom series	High-resolution digital RGB camera	1.66	36	6 × 5	Mean, median, standard deviation, coefficient of variation, and 25th, 50th, 75th, and 95th percentiles	0.89	6
Schirrmann et al. (2016)	GS41 to GS83	P-Y6, hexapilots, dresden (hexacopter)	Sony NEX 7 24 mgpx	1.2	20	1 x 1	90th percentile	0.76–0.92	6.0–15.0
Song and Wang (2019)	GS31, GS65 and GS83	DJI phantom 3	High-resolution digital RGB camera	1.5	15	2 × 2	Cuboid filter 3D classification	–	4.5–7.7
Yuan et al. (2018)	GS30 to GS87	Matrice 600 pro (M600) and DJI phantom 3 Pro	High-resolution digital RGB camera	0.47–0.67	100	1.5 x 1.524	89th and 100th percentiles	0.91	9
Yue et al. (2017)	GS30 and GS65	DJI S1000	UHD 185 firefly—hyperspectral sensor^#^	1	48	6 × 8	Average of pixel values	0.69	19

**GS, Growth stage; UAV, unmanned aerial vehicles; GSD, ground sampling distances (cm/pixel). ^#^Adapted sensor capturing 450–950 nm wavelengths. ^$^30 and 19 mm focal length was used to equip the camera. ^‡^Coefficient of determination (R^2^).****^¶^****Root mean square error (RMSE) between PHground and PHaerial, where the single numbers are the joining values obtained across GS by the respective authors.**

Despite the advances of this method for estimating PH using UAV imagery (PHaerial), there are several factors that should be taken into account, such as image ground sampling distance (GSD) or weather conditions, that can potentially affect its performance and accuracy (Han et al., 2018; Lu et al., 2019). Willkomm et al. (2016) also found that plant movement during image acquisition and the lack of protocol for field hand measurements can lead to PH underestimations. In view of these limitations, an effective and low-cost workflow using RGB camera can be deployed considering an adequate GSD and statistical aerial analysis under field conditions.

To the best of our knowledge, the image and data quality of ground-truth measurements (PHground) have not been adequately evaluated to assess their impact on PHaerial at individual plot growth stages (GS) in wheat breeding programs. Therefore, this study aims to validate PH derived from RGB imagery data and to understand the effect of data quality from different UAV platforms and PHground. The study’s specific objectives are: (i) to develop a semi-automated low-cost workflow for extraction, analysis, and evaluation of PHaerial at multiple GS; (ii) to compare different UAV platforms used for PH estimations, and; (iii) to assess the potential environmental issues associated between PHground and PHaerial. Finally, we investigate PHaerial and PHground predictions using the genotypic values.

## Materials and Methods

### Plant Material, Site Description, and Data Collection

The experiments were conducted over two spring wheat (*Triticum aestivum* L.) growing breeding cycles: 2016–2017 and 2017–2018 at the CIMMYT experimental station Campo Experimental Norman E. Borlaug in Ciudad Obregon, northwestern Mexico (27°20’N; 109°54’W; and 38 masl). Environmental and management details of this site are given in Sayre et al. (1997). Two spring wheat panels were studied under potential yield conditions: the high biomass association panel (HiBAP)-I and the HiBAP-II. Fifty inbred lines were used for the validation in HiBAP-I during the 2016–2017 (Y17) and 2017–2018 (Y18) crop cycle, while the whole population of 150 lines was measured in HiBAP-II during the 2017–2018 (Y18). Both panels include representative lines derived from breeding and pre-breeding programs with a restricted range of maturity and height (Molero et al., 2019). The experimental design in both HiBAP panels consisted of an alpha-lattice design with two replicates and 30 incomplete blocks per replicate. The plots consisted of two beds in HiBAP-I Y17 and one bed in HiBAP I Y18 with two plant rows on the top of the beds for both trials. In HiBAP-II Y18, three replicates were evaluated in two beds plots. The beds in all three trials were 0.8 m wide, while the inter-row spacing within the bed and the space between beds were 0.24 and 0.36 m, respectively. Plot length was 4 m for HiBAP I Y17 and HiBAP II Y18 and 2 m for HiBAP I Y18.

Aerial (PHaerial, using UAV platforms) and ground-truth (PHground) plant height (PH) phenotyping were performed in the experiments during the following GSs: 40 days after emergence (E+40), at booting (B), 7 days after anthesis (A+7) and at physiological maturity (M). PHground was measured using a ruler when 50% of the plot reached a particular GS, as described by Torres and Pietragalla (2012). Similarly, the two UAVs were flown on the same day or 1 day apart, depending on the logistics in the field and the weather conditions. The optimal time and weather conditions for UAV data collection were defined as: around solar noon, under clear sky, and a low wind speed. A summary of solar radiation and wind speed conditions during the entire flight campaigns for each platform used is given in Supplementary Table S1. The average height was obtained from four random individual culms inside each plot (two in each bed), measuring the distance from the soil surface to the tip of the spike, excluding the awns, and avoiding any mounds or cracks in the soil.

### Flight Campaign and Imagery Quality Parameters

The flight campaigns were performed with a high-resolution digital RGB camera mounted in two different types of UAVs across the growing cycles: the fixed-wing (*Plat*_*FW*_)eBee (SenseFly Ltd., Cheseaux-Lausanne, Switzerland) employed in Y17 and Y18, the multi-rotor (*Plat*_*MR*_) AscTec Falcon 8 (Ascending Technologies, Krailling, Germany) in Y17 and the Matrice 100 (DJI, Nanshan, Shenzhen, China) in Y18.

The flights were planned at the time of PHground phenotyping for assessing trials according to the predominant GS of interest in this study (E+40, B, A+7, and M). Table 2 summarizes the number of flights and main specifications for each GS assessment at the time of PHaerial estimation, including the number of flights. The ground control points (GCPs) or post-processed kinematic (PPK; see below for details) were used for georeferencing corrections. A set of black and white squared GCPs were uniformly distributed over the entire field area in all trials. These GCPs, distributed for each panel according to Table 2, were surveyed with a Global Navigation Satellite System (GNSS) receiver using a real-time kinematic (RTK) correction (Trimble R4 GNSS system, Trimble, Sunnyvale, CA, United States). Additionally, 11 checkpoints (CP), surveyed using RTK correction, were placed across the site during the crop cycle Y18 for georeferenced accuracy assessment of the orthomosaics.

**TABLE 2:** Crop phenology information across the measurements presented as days after emergence (DAE), the predominant development crop stage expressed by Zadoks growth scale, corresponding phenological stage and identification nomenclature in this investigation, as well as the number of flights for each platform using ground control points (GCPs) or post-processed kinematic (PPK) corrections for fixed-wing (*Plat*_*FW*_) and multi-rotor (*Plat*_*MR*_) platforms.

**Trial**	**Pred. phenological stage**	**Ident. stage^a^**	**Zadoks scale^b^**	**DAE^c^**	**Number of flights (Plat_FW_)**		**Number of flights (Plat_MR_)**
					**PPK**	**GCP**	**Only GCP**
**HiBAP-I Y17** (30/Nov/2016^d^)	Stem elongation	E+40	37–39	40	1	0	1
	Flowering	A+7	61–65	73–87	5	0	5
	Maturity	M	91–92	100	1	0	1
**HiBAP-II Y18** (03/Dec/2017)	Stem elongation	E+40	37–39	40	1	0	0
	Booting	B	41–47	55–72	6	0	7
	Flowering	A+7	61–69	76–98	6	2	8
	Maturity	M	91–92	105–118	2	0	3
**HiBAP-I Y18** (18/Dec/2017)	Stem elongation	E+40	37–39	40	1	0	1
	Booting	B	41–45	55–69	2	1	3
	Flowering	A+7	61–69	74–91	1	1	2
	Maturity	M	91–92	106–111	1	0	1

**^*a*^Specific identification of the GS estimated/predominant: at 40 days after emergence (E+40), booting (B), 7 days after anthesis (A+7) and at physiological maturity (M). ^*b*^The decimal (or Zadoks Scale according to Zadoks et al., 1974) growth stage code estimated according to the genetic variability. ^*c*^Days after emergence represented by 50% of the plants with the first leaf through coleoptile (GS10). ^*d*^Emergence date in each crop season.**

The flights of the *Plat*_*FW*_ followed the technical recommendations in Loladze et al. (2019) and are described in Table 3. The flight plan was designed for north/south and east/west flights to achieve both a lateral and longitudinal overlap of 80%. The flights covered an area larger than the experiment to cover the entire experimental field and obtain accurate orthomosaics. High-accuracy corrections of the geolocation data measured with the *Plat*_*FW*_ global navigation satellite system (GNSS) were calculated in the post-processing stage using the position of a fixed base station as a reference and the PPK correction while imagery geotagging (Benassi et al., 2017; Forlani et al., 2018).

**TABLE 3:** Parameters of flight specifications details for fixed-wind (*Plat*_*FW*_) and multi-rotor (*Plat*_*MR*_) platforms.

	**HiBAP-I Y17**		**HiBAP-II Y18**		**HiBAP-I Y18**	
	**Plat_FW_**	**Plat_MR_**	**Plat_FW_**	**Plat_MR_**	**Plat_FW_**	**Plat_MR_**
Sensor	Canon PowerShot 110 camera of 16.2 MegaPixels	Sony NEX 5	SODA	ZenMuse X5	SODA	ZenMuse X5
Resolution (image pixels)	4,608 × 3,456	4,592 × 3,056	5,472 × 3,648	4,608 × 3,456	5,472 × 3,648	4,608 × 3,456
GSD^a^ resolution (cm/Pixel)	1.7	0.7	1.7	0.7	1.7	0.7
GCPs^b^ numbers for internal processing	7	7	9	9	7	7
Flight altitude	65	30	85	30	85	30

**^*a*^GSD, ground sampling distance. ^*b*^GCPs: ground control points used for internal processing forPlat*_*MR*_*andPlat*_*FW*_*without PPK corrections.**

The flight plans for both multi-rotor platforms were designed to achieve lateral and longitudinal overlaps of 80%, flying north/south. The flight operations of these multi-rotor UAVs are shown in Table 3, and further details can be checked in Tattaris et al. (2016) for the AscTec Falcon 8, and in Horton and Ranganathan (2018) for the Matrice 100. The flight plans of both types of platforms, *Plat*_*FW*_ and *Plat*_*MR*_, were designed to acquire images with different ground sampling distances (GSD in Table 3).

### Three-Dimensional Crop Reconstruction and Plant Height Accuracy Assessment

**FIGURE 1:**
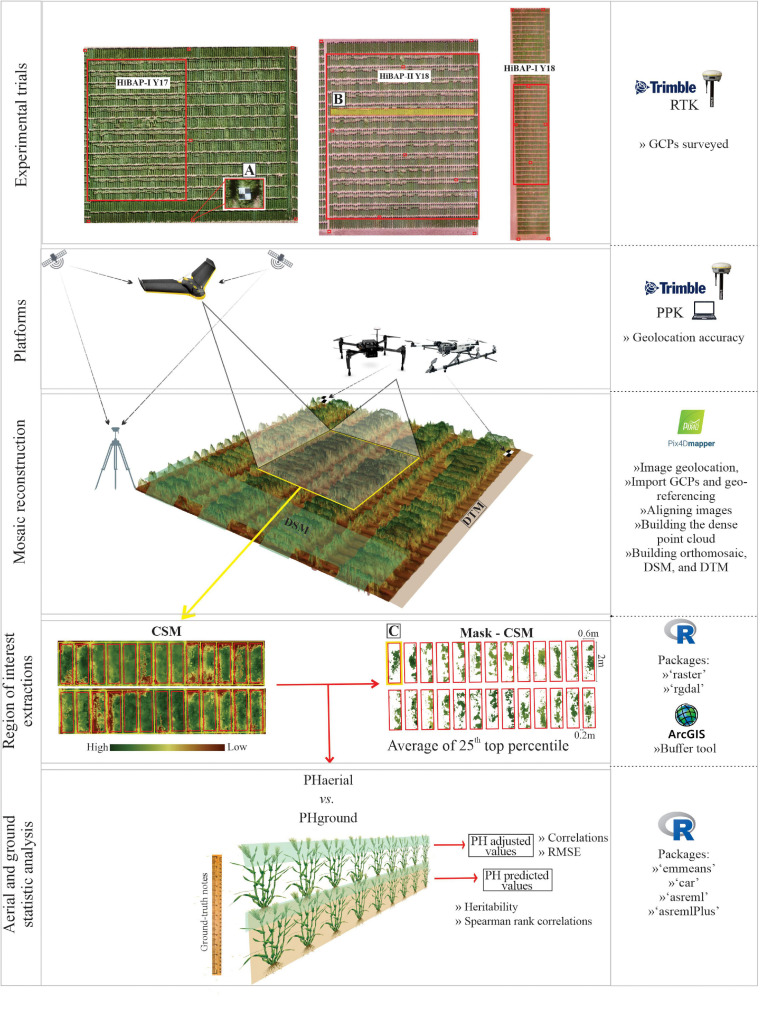
Phenotyping workflow for estimation of plant height (PH) using *Plat*_*FW*_and *Plat*_*MR*_ (UAVs) platforms (PHaerial) used in north-west Mexico during the 2017 and 2018 growing cycles. PHground, ground-truth measurements; DTM, digital terrain model; DSM, digital surface model; CSM, crop surface model. **(A–C)** are GCPs design, locality of the profile selected, and cropped area with the mask of CSM as the top 25th percentile pixels value, respectively.

The aerial data collected by both types of platforms were geotagged for orthomosaic processing using Pix4D Mapper software (v4.4.10; Pix4D, Lausanne, Switzerland). Images were imported into Pix4D software. GCPs were manually located to improve the accuracy of the three-dimensional (3D) point cloud georeferencing for *Plat*_*FW*_ flights that did not use PPK corrections, as well as for the flight campaign using *Plat*_*MR*_ (Figure 1A). The georeference accuracy was checked by rather than in the bundle adjustment of the orthomosaic product. The digital terrain model (DTM, i.e., the topography of the site without any plant) was generated for each trial from images collected by a single flight of each UAV platform prior to the vegetation emergence. The digital surface model (DSM; i.e., the topography of the site accounting for the plants) was obtained along with vegetation development at each GS.

The DSM and DTM rasters were computed following the workflow recommended by Pix4D for high-resolution RGB imagery (Pix4D, 2019b). This workflow uses a structure from motion (SfM) algorithm (Ullman, 1979; Snavely et al., 2008) to obtain a 3D point cloud. The point cloud was later meshed via an algorithm based on Delauney triangulation (Matthew et al., 2009; Susanto et al., 2016) computed on multiple image scales with noise filtering and a “sharp” surface smoothing filter. Afterward, the DTM was subtracted from the DSM to estimate the crop surface model (CSM, i.e., the height of individual plot surfaces) using R software version 3.6.1 (R Core Team, 2018). The PHaerial scripts used to perform the image analyses and trait extract are available at https://github.com/volpatoo/HTP-via-drone-imagery/tree/master/UAV-HTP_PlantHeight. Figure 1 represents the major steps of the data acquisition and processing, as well as the software, packages, and tools used in this workflow.

Before computing the CSM for all flight campaigns, we conducted a preliminary test to define the best parametrization of the Pix4D workflow. Different settings in Pix4D were combined and tested to obtain the best high-density point clouds and DSM (for details, see Supplementary Table S1). The test results (not shown) were compared based on the accuracy of PHaerial against the PHground for each platform. This exercise used the data from the Y17 growing cycle at E+40, A+7, and M GS and the best performing processing scheme parameters to generate the 3D point clouds of all the flight campaigns (Supplementary Table S1).

The PH from the CSM raster was assessed using ArcGIS (version 10.6, Esri Inc., Redlands, United States). The buffer tool was used to create regions of interest (*ROI*_*PH*_) to extract PH values from each plot (Figure 1C). Plant breeding trials usually consist of small plots within 0.5–1 m of each other in the interests of trial uniformity. Under these conditions, the canopies of adjacent plots can interfere with one another by shading, lodging, or wind load. The small plots can easily cause noise in the PH estimation, especially after flowering. To ensure the extraction of pure pixel values (i.e., pixels containing only information from the plot of interest), we built the regions of interest with a buffer zone of 0.1 m from the plot edges aligned at the center of the two-bed rows. *ROI*_*PH*_ were exported as polygons into a shapefile for the data extraction.

We extracted data from the regions of interest by overlapping the CSM and the shapefile containing the *ROI*_*PH*_ using the R packages “raster” and “rgdal.” Average PHaerial was calculated for each plot using pixel values greater than the 75th percentile for that plot. We tested different criteria for selecting pixels within the *ROI*_*PH*_ but this proved to be the optimum indicator for PHaerial based on comparison with the PHground values.

### Accuracy Assessment of Orthomosaics Georeferencing

**FIGURE 2:**
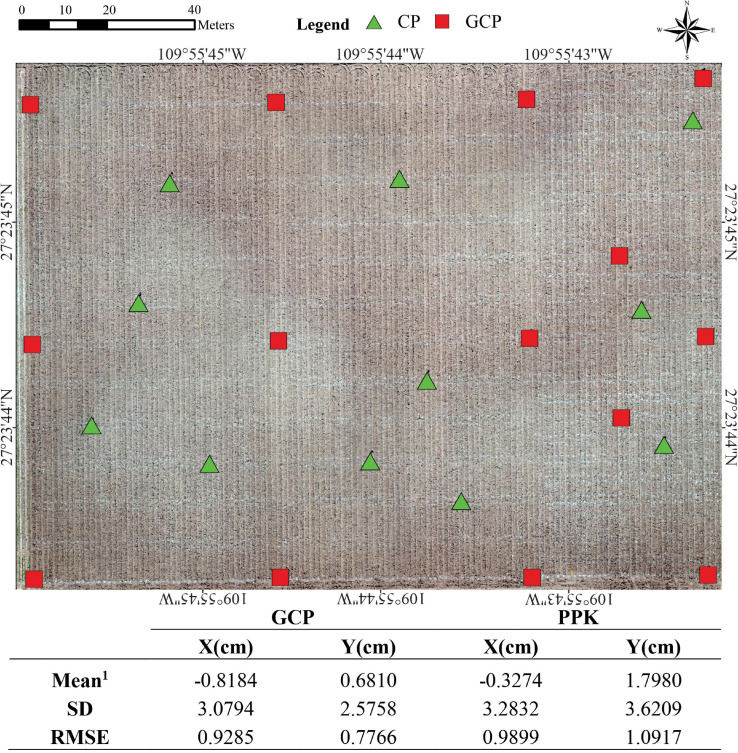
The mean delta-X and delta-Y from all geocoordinates obtained from 11 checkpoints (CP) vs. the geocoordinates obtained from the orthomosaic (CSM) resulted from 14 GCP and PPK corrections. Standard deviation (SD) and Root Mean Square Error (RMSE) for X and Y coordinates. The base image corresponds to the bare soil flight using *Plat*_*FW*_in the HiBAP-II trial during November 2018. ^1^Mean difference between measured coordinates to GCP and PPK.

The automation of data extraction per plot requires a high accuracy in the orthomosaics and DTM georeferencing. To ensure this, we performed a preliminary study using two techniques: GCPs and PPK correction. PPK correction was used to obtain accurately geotagged *Plat*_*FW*_ imagery. The table in Figure 2 shows the comparison between the absolute accuracy of longitude and latitude coordinates estimated by the two methods. The accuracy is expressed as the difference between the XY geocoordinates from the CPs (which were not used in the bundle adjustment process) by comparing the coordinates of the CPs obtained at the CSM with the in-site geocoordinates obtained by an RTK GNSS system (i.e., delta-X and delta-Y, being X latitude and Y longitude). The delta-X and delta-Y were calculated for both platforms using the set of 11 CPs placed in the field during the crop cycle Y18. Additionally, the root mean square error (RMSE) of the differences between X and Y coordinates, the mean values and the standard deviations (SD) were computed. These parameters showed that the PPK achieved similar results than those obtained with GCPs for horizontal XY coordinates (RMSE ∼ 1 cm and SD < 3.62 cm; Figure 2). The average accuracy measured as SD on the CPs coordinates was in agreement with the accepted limits mentioned by Vautherin et al. (2016): one to two times the GSD in X and Y directions either to GCP or PPK corrections.

**FIGURE 3:**
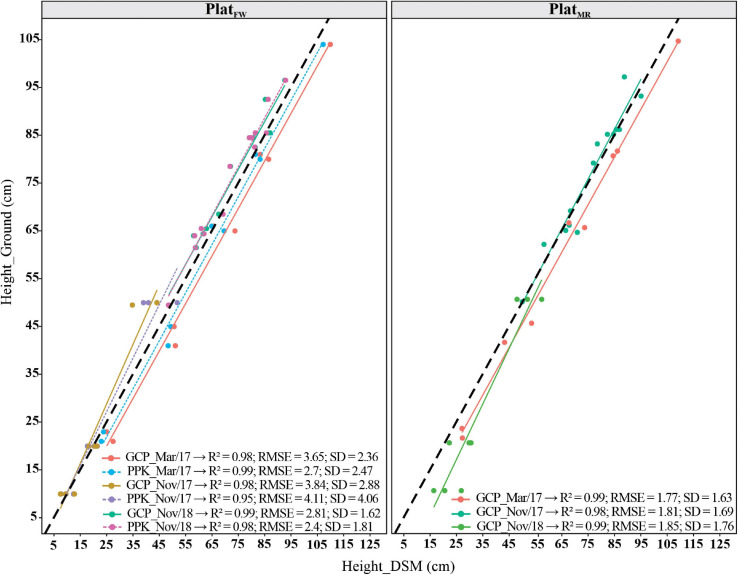
Coefficient of determination (*R*^2^), root mean square deviation (RMSE), and standard deviation (SD) of the errors, between ground control points (GCPs) height estimate from the digital surface model (DSM), and ground-truth measurements for three different dates of flights using PPK for fixed-wind platform (*Plat*_*FW*_) and only GCP corrections for *Plat*_*FW*_ and multi-rotor (*Plat*_*MR*_). The dark black dashed diagonals represent the 1:1 line, and the colored solid (GCP) and dashed (PPK) lines represent the regression lines.

We also evaluated the accuracy for altitude estimations (i.e., *Z*-axis) by contrasting calculated and ground-truth GCP height values using one flight in each breeding cycle for *Plat*_*FW*_ and *Plat*_*MR*_ (Figure 3). The height accuracy measured on the GCPs was acceptable in all flight dates, with the *Plat*_*MR*_ showing slightly better results (RMSE = 1.77–1.85; and *SD* = 1.63–1.76) than *Plat*_*FW*_ (RMSE = 2.81–3.84; and *SD* = 1.62–2.88). The *R*^2^ was greater than 0.95 for all cases. The accuracy measured as SD also followed the criterion adopted by Vautherin et al. (2016): two to three times the GSD in the Z direction for both platforms. Overall, the accuracy obtained in the CSMs using PPK and GCP approaches reached similar results.

### Statistical Models and Genetic Selection Evaluation

Since we conducted few flights at each GS, we first built a linear model to fit a single PH value per plot. In this model, the PHaerial from each platform at each GS was used as a dependent variable against plot and number of flights as explanatory variables. The adjusted means per plot for PHaerial and PHground values were then used to calculate best linear unbiased estimates (BLUEs) within each crop cycle, using the following model:

$$y_{i\,j\,k}=\mu +g_{i}+r_{j}+b_{k\,\left(j\right)}+\varepsilon_{i\,j\,k}$$

where *y*_*ijk*_ is the trait value for genotype *i*, replicate *j*, and block *k*; *μ* is the overall mean; *g_i* is the fixed effect for genotype *i*; *r_j* is the random effect for replicate *j*, which are assumed to be independently and identically distributed according to a normal distribution with mean zero and variance $\sigma_{r}^{2}$; that is, $r_{j}∼i\,i\,d\,N\,\left(0,\sigma_{r}^{2}\right)$; $b_{k\,\left(j\right)}∼i\,i\,d\,N\,\left(0,\sigma_{b}^{2}\right)$ is the random effect for block *k* within replicated *j;* and $\varepsilon_{i\,j\,k}∼i\,i\,d\,N\,\left(0,\sigma_{\varepsilon }^{2}\right)$ is the residual effect.

For each growing cycle, Pearson’s correlations, *R*^2^, and RMSE for PHs between PHground and PHaerial were calculated using the BLUEs derived from the above model at each GS after removing the outliers. Outliers were flagged using studentized residual from PHground values, and the significance of their correlation with PHaerial was determined by the Bonferroni test at *P* < 0.01 (Fox and Weisberg, 2019). The identified outliers were removed from both PHground and PHaerial to perform the analysis. Finally, the *RMSE*_*dev*_ was computed to measure the deviation between the estimated values (PHaerial) and the measured values (PHground) across GS in each trial, according to Zhou et al. (2020).

The validation of the prediction model used best linear unbiased predictions (BLUPs) and heritability for PHground and PHaerial. The genotypic variance components ($\sigma_{g}^{2}$ and $\sigma_{\varepsilon }^{2}$) were derived by the fitted model described above for both PHground and PHaerial to calculate the broad-sense heritability ($H_{g}^{2}$, sometimes termed “repeatability”) with the genotype *g_i* treated as a random effect in which $g_{i}∼i\,i\,d\,N\,\left(0,\sigma_{g}^{2}\right)$. Thus, $H_{g}^{2}$ quantifying the repeatability of the plant height trait estimation was computed as the ratio between the genotypic to the total variances (Holland et al., 2002). The significance (Ripley, 2019) of the Spearman rank correlation coefficient (ρ) (Spearman, 1904) was calculated using the BLUPs from both UAV-platforms against PHground for assessing the accuracy of genotypic rank selection.

Additionally, we measured 50 coincident genotypes in Y17 and Y18 (HiBAP-I) crop cycles (considering $g_{i}∼i\,i\,d\,N\,\left(0,A\,\sigma_{g}^{2}\right)$ where ***A*** is the associated additive relationship matrix) to obtain the narrow-sense heritability $\left(h_{a}^{2}\right)$ for both UAV-platforms and to assess the accuracy under a G × E interaction model design, including the genotype × year interaction effect (*t*_*ge*_) also as random with $t_{g\,e}∼N\,\left(0,\sigma_{g\,e}^{2}\right)$. The data collected during booting in HiBAP-I Y18 was removed from the statistic-genetic model for G × E interaction in order to match better the GSs and calculate the BLUPs. For this analysis, *R*^2^ represents the accuracy of predicted values from the correlations between the PHground and PHaerial. The standard errors (SE) of the heritability parameters in both validation models were obtained through mixed model output (Wolak, 2018).

We used the R software to run the statistical analyses, including linear models (Gilmour et al., 2015), multiple comparison procedures (Lenth, 2016), mixed and prediction models (Brien, 2018), and testing of model terms (Fox et al., 2019). The coefficients of parentage for the pedigree relationship matrices (*A*) were estimated as twice the coefficient of parentage using the “Browse” application within the International Crop Information System software package (McLaren et al., 2000).

## Results

### Descriptive Statistics Across Growth Stages

**FIGURE 4:**
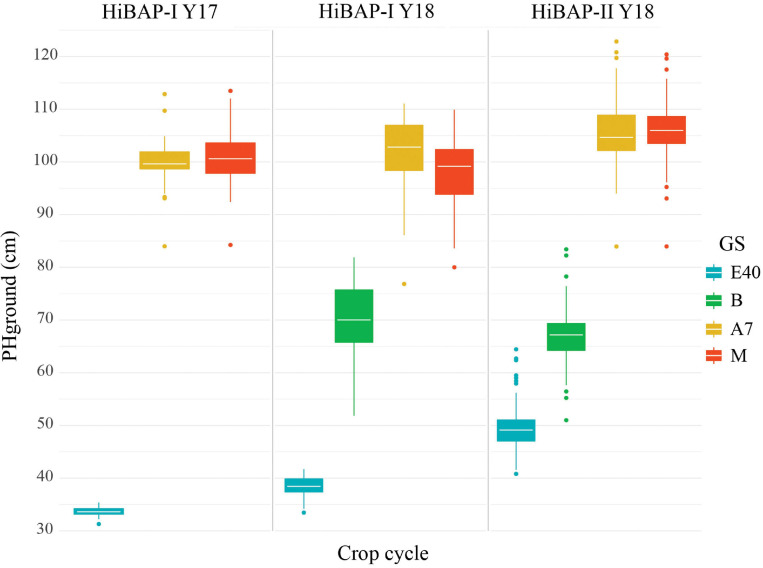
Boxplot of BLUEs for wheat plant height (PH, cm) in each of the observed crop cycles at the following growth stages (GS): 40 days after emergence (E+40), booting (B), 7 days after flowering (A+7) and at physiological maturity (M).

PHground values were similar across crop cycles at the same evaluated GS (Figure 4). The heterogeneity within each trial remained relatively stable at B, A+7, and M (*SD* = 4.13–4.97 in HiBAP-I Y17, *SD* = 6.02–7.04 in HiBAP-I Y18, and *SD* = 4.62–5.65 in HiBAP-II Y18). The median value and SD for ground-truth PH measured at E+40 showed some discrepancies across cycles and trials, possibly attributable to the different genotypes used in each HiBAP panel, the year effect and differences in emergence dates.

### UAV Plant Height Assessment and Validation

**FIGURE 5:**
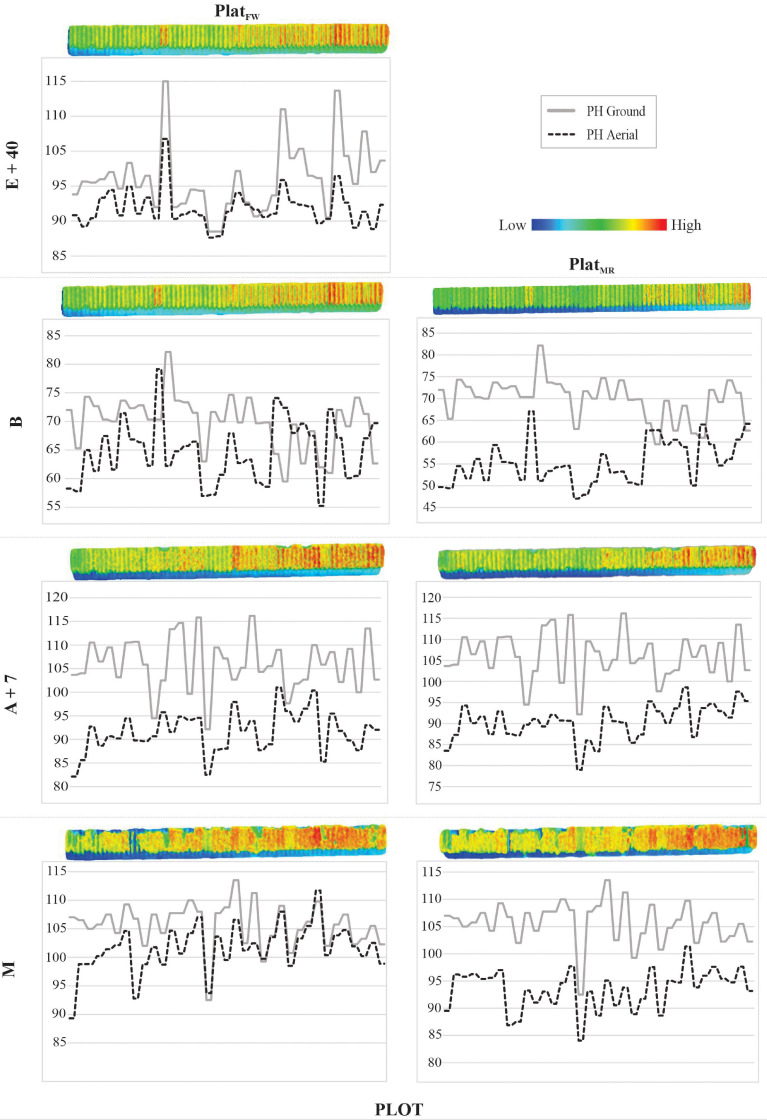
Profile of 70 plots from HiBAP-II for *Plat*_*FW*_ and *Plat*_*MR*_ at the following growth stages (GS): 40 days after emergence (E+40), booting (B), 7 days after flowering (A+7), and at physiological maturity (M). Plant height (PH, cm) via ground-truth (ground) and unmanned aerial vehicle (UAV) data (aerial) are represented in the solid and dotted lines, respectively, matching with low (blueish) and high (reddish) color scale to PH. The 2D plots profiles image were generated using the textured mesh feature via the densified point cloud of Pix4D processing.

The PHaerial estimates were, in general, similar to PHground values. This matching can be visualized in Figure 5, where transects of PHground and PHaerial data from HiBAP-II are compared (refer to Figure 1B for the location of this transect within the HiBAP-II trial). Considerable mismatching between PHground and PHaerial values was detected at booting (B), whereas the best agreement was observed during maturity (M).

**FIGURE 6:**
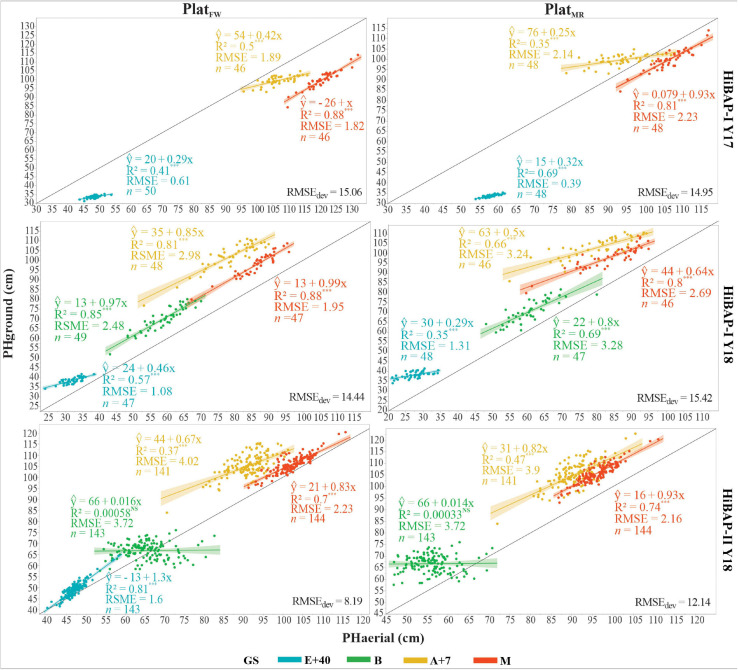
Linear relationship between plant height (PH, cm) estimated from the unmanned aerial vehicle (UAV-based) data (PHaerial) and that ground-truth measured manually (PHground), as well as RMSE, and the number (*n*) of genotypes considered at four growth stages (GS): 40 days after emergence (E+40), booting (B), 7 days after flowering (A+7), and at physiological maturity (M) for *Plat*_*FW*_ and *Plat*_*MR*_considering all locations in this study. Black solid line shows the 1:1 lines; light shadow color represents a 99% confidence interval. The *RMSE*_*dev*_ in the bottom right represents the deviation between the PHaerial and the PHground across GS. ^∗∗∗^ indicate *p*-value of the coefficient of determination (*R*^2^), with ^∗∗∗^*P* < 0.001; NS, non-significative value.

The agreement between PHground and PHaerial data was further confirmed by the strong correlations observed during most of the growing cycle for both platforms (Figure 6). The coefficient of determination at the different GS ranged from non-significant to *R*^2^ = 0.88, and from non-significant to *R*^2^ = 0.81, for *Plat*_*FW*_ and *Plat*_*MR*_, respectively. The measurements at maturity showed the highest and most consistent correlations across the different trials and platforms, with *R*^2^ values ranging between 0.7 and 0.88. The lowest correlations were obtained at booting, observing even non-significance in HiBAP-II Y18 for both *Plat*_*FW*_ and *Plat*_*MR*_ (*p* ≥ 0.78). The *Plat*_*FW*_ platform performed better than *Plat*_*MR*_ in HiBAP-I, except at E+40 during Y17, where *Plat*_*MR*_showed a coefficient of determination of 0.66 against 0.41 in *Plat*_*FW*_. Conversely, *Plat*_*MR*_ performed slightly better than *Plat*_*FW*_ in HiBAP-II (Figure 6), particularly at A+7 (*R*^2^ = 0.47 in *Plat*_*MR*_ vs. *R*^2^ = 0.37 in *Plat*_*FW*_) and M (*R*^2^ = 0.74 in *Plat*_*MR*_ vs. *R*^2^ = 0.7 in *Plat*_*FW*_). Overall, the RMSE of the predicted model for individual GS did not exceeded 4.02 cm. However, the *RMSE*_*dev*_ obtained across GS for each platform in HiBAP-I Y17 and HiBAP-I Y18 were around 15 cm (*RMSE*_*dev*_ = 15.06 and 14.95 cm in HiBAP-I Y17; *RMSE*_*dev*_ = 14.44 and 15.42 in HiBAP-I Y 18). The best performance for *RMSE*_*dev*_ was in HiBAP-II Y18 in both platforms. Nevertheless, the *Plat*_*FW*_ provided better results than *Plat*_*MR*_ (*RMSE*_*dev*_ = 8.19 for *Plat*_*FW*_ *vs.* 12.14 = *Plat*_*MR*_).

### HTP for Genotypic Prediction of Plant Height From Wheat Breeding Trials

**FIGURE 7:**
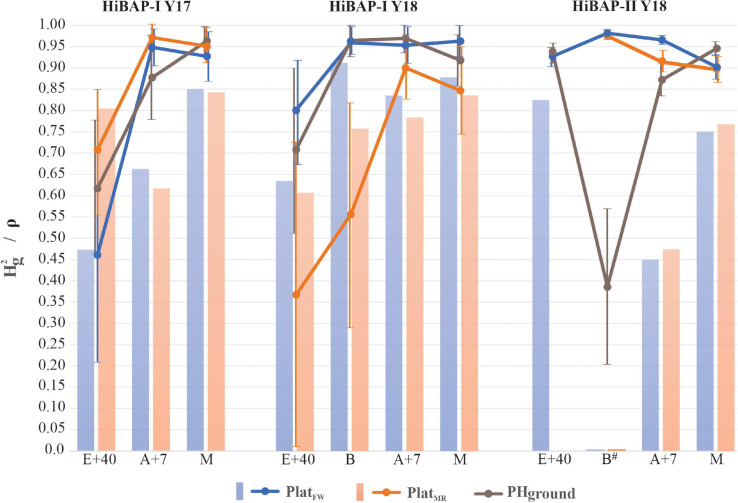
The solid color lines represent the broad-sense heritability ($H_{g}^{2}$) across crop cycles (2016–2017 and 2017–2018) for *Plat*_*FW*_,*Plat*_*MR*_ and PHground in the growth stages: 40 days after emergence (E+40), booting (B), 7 days after flowering (A+7), and at physiological maturity (M) and its 95% confidence interval based on standard errors. The color bars show the Spearman Rank Correlation (ρ) from the predicted values between PHground and PHaerial. All ρ significant at *P* < 0.0001 except to B (non-significant) in HiBAP-II Y18.

The evaluation strategy using $H_{g}^{2}$ shows strong potential for PHaerial implementation in a wheat breeding program, as PHaerial reached similar or higher values $H_{g}^{2}$ than those from PHground for each GS and across locations (Figure 7).

The *Plat*_*MR*_ provided better $H_{g}^{2}$ estimations than *Plat*_*FW*_ and PHground for all GS at HiBAP-I Y17, except for maturity from PHground, ranging from 0.71 to 0.97 for *Plat*_*MR*_ vs. 0.46–0.93 for *Plat*_*FW*_, and vs. 0.62–0.96 for PHground. On the other hand, the *Plat*_*FW*_ obtained greater $H_{g}^{2}$ values than *Plat*_*MR*_ and PHground at HiBAP-I Y18 in all GS analyzed, except for A+7 from PHground ($H_{g}^{2}$ = 0.80, 0.96, 0.95, and 0.90 for *Plat*_*FW*_ vs. $H_{g}^{2}$ = 0.37, 0.56, 0.90, and 0.85 for *Plat*_*MR*_, and vs. $H_{g}^{2}$ = 0.71, 0.96, 0.97, and 0.92 for PHground, for E+40, B, A+7 and M, respectively). On HiBAP-II Y18, both platforms obtained similar results, but $H_{g}^{2}$ PHaerial estimations were significantly better than PHground at booting and *Plat*_*FW*_ at A+7 was better estimations than *Plat*_*MR*_ and PHground. Furthermore, *Plat*_*FW*_ and PHground in HiBAP-I Y18 provided more accurate estimations of $H_{g}^{2}$ in comparison with *Plat*_*MR*_ at booting.

Overall, the $H_{g}^{2}$ responses were in agreement with the results from the correlations (*R*^2^) between PHground and PHaerial data. For HiBAP-I trials the $H_{g}^{2}$ degraded at E+40, and increased at later GS. Additionally, the UAV platforms showed better $H_{g}^{2}$ estimations than PHground across GS for each crop cycle ($H_{g}^{2}$ 0.78, 0.92 and 0.94 for *Plat*_*FW*_, and $H_{g}^{2}$ 0.88, 0.67 and 0.93 for *Plat*_*MR*_ vs. $H_{g}^{2}$ 0.82, 0.89 and 0.78 for PHground, within HiBAP-I Y17, HiBAP-I Y18, and HiBAP-II Y18 trials, respectively).

The Spearman rank correlations (ρ) between predicted values for PHaerial and PHground were significant (*P* < 0.001) at all GS in all trials except at booting in HiBAP-II Y18. The highest ρ for HiBAP-I Y17 was observed at maturity for both platforms, and at HiBAP-I Y18, except at booting using the *Plat*_*FW*_ (ρ = 0.91). Moreover, the greatest ρ in HiBAP-II Y18 was achieved at E+40 via *Plat*_*FW*_ (ρ = 0.83). Lower, but still significant correlations using both platforms were observed at A+7 in HiBAP-II Y18 (ρ = 0.45 for *Plat*_*FW*_, and ρ = 0.46 for *Plat*_*FW*_) (Figure 7).

**FIGURE 8:**
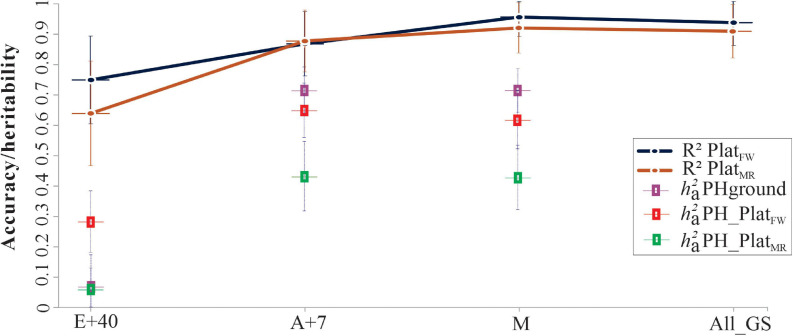
Narrow-sense heritability ($h_{a}^{2})$ and coefficient of determination (*R*^2^) from genotype-by-environment interaction (G × E) model using HiBAP-I genotypes measured in 2016–2017 and 2017–2018 growing cycles via *Plat*_*FW*_ and *Plat*_*MR*_, and across growth stages (GS): 40 days after emergence (E+40), 7 days after flowering (A+7), and at physiological maturity (M). The solid color lines represent the accuracy (*R*^2^) for predict values between PHground and PHaerial, plus the confidence interval (CI) by the error bar. The colored squared represents the $h_{a}^{2}$ and the error bars indicate standard error. All growth stages (All_GS) were also considered to confirm the accuracy of genetic correlations. The cross lines give the upper and lower bounds of the 95% CI of the correlations computed using $\hat{\rho }=1.96\,\sqrt{\left(1-\hat{\rho }\right)/\left(n-2\right)}$, where $\hat{\rho }$ is the estimated correlation, and *n* is the number of records used to compute the correlation.

When genotype-environment interaction (G × E) effects were considered in the prediction of the genotypic PH values, the narrow-sense heritability ($h_{a}^{2}$) for *Plat*_*FW*_ was greater than for *Plat*_*MR*_ for all GS analyzed ($h_{a}^{2}$ 0.29, 0.65, and 0.62 in *Plat*_*FW*_, vs. $h_{a}^{2}$ 0.06, 0.42, and 0.41 in *Plat*_*MR*_, for E+40, A+7, and maturity growth stages, respectively). However, the $h_{a}^{2}$from PHground was higher than PHaerial at A+7 ($h_{a}^{2}$ 0.71) and M ($h_{a}^{2}$ 0.71). The accuracy (or *R*^2^) remains constant across GS ranging from 0.75 to 0.96 in *Plat*_*FW*_ vs. 0.64–0.92 in *Plat*_*MR*_, but with lower values at E+40 for both UAV platforms (Figure 8).

## Discussion

The present study aimed to prove the applicability of aerial photogrammetry (i.e., using UAVs) to estimate PH in the wheat breeding context. Throughout the growing season, aerial HTP approaches were conducted on two different wheat panels (HiBAP-I and II) with two UAV platform classes (multi-rotor and fixed-wing) equipped with RGB cameras using PPK or GCP corrections. Our findings showed that for most of the growing stages, the UAV-based data (PHaerial) could be used for reliable estimations of PH and that genotype selection based on this data was equivalent to that obtained by manual ground measurements (PHground) (*R*^2^ = 0.35–0.88). We attribute the good results obtained for a large number of experimental wheat plots (100 for HiBAP-I and 450 for HiBAP-II) partly to the strategy used in the pixel PH values extraction within each plot. The selection of pixels from the top 25th percentile within each plot region of interest of the imagery was intended to increase the proportion of observations from the upper canopy in further analyses (Figure 1C). This strategy showed the best results in our study, differing from other studies, as shown in Table 1, which used either the average or very restricted statistical methods such as the 99th or 99.5th percentiles. These restricted models may be “too selective”—going against field hand measurement protocols where randomization within the plot and a minimum number of measurements should be respected.

### Assessing the Data Quality of UAV-Based Plant Height Estimations

A number of factors can have an impact on PHaerial estimations. In this section, we outline major potential sources of error discussed in previous studies, including UAV imagery parameters, choice of platform, and environmental factors, and their potential relevance to our study.

Ground sampling distance (GSD) is important in creating high-quality orthomosaics via orthorectification to obtain the DSM from the dense image matching as an additional source of data to enhance the PH model accuracy (Madec et al., 2017; Lu et al., 2019; Wilke et al., 2019). This orthomosaic generation method confers more accurate 3D points due to the extraction of common characteristic points (keypoints) in different images and by removing perspective distortion from the images using the DSM (Pix4D, 2019a). In our study, the differences observed in the performance of the two aerial platforms suggest that UAV imagery parameters such as GSD, altitude, and point cloud density may have influenced the PHaerial estimation accuracy. Our results indicate the *Plat*_*FW*_ produced slightly more accurate PH estimations using a higher GSD (GSD = 2 cm/pixel) and a lower point cloud density (see Supplementary Table S1) compared to *Plat*_*MR*_. Therefore, our results suggest there is no need to work with sub-centimeter resolution for DSM reconstruction when targeting PH estimation. This is in alignment with results obtained by Lu et al. (2019).

Even though in the case of our study *Plat*_*FW*_ produced the best correlations and RMSE results overall, the choice between the two classes of platforms that have been evaluated in this study depends on the processing pipeline used, plant breeding target, and several other technical factors such as area extension, pilot expertise, total flight time, and intended GSD (Puri et al., 2017; Park et al., 2019). Each of these decisions can affect the quality of the PH data.

Other authors have noted several sources of error in aerial estimation of PH in wheat crops, including inefficient image pre- and post-processing due to suboptimal flying altitude, inaccurate DTM construction and errors in height extraction strategy from images (Hassan et al., 2019a). Chu et al. (2017) demonstrated that under unfavorable weather conditions, the quality of a dense point could affect the 3D-pixel constructions. For example, wheat PH data bias can occur due to wind conditions when using high spatial resolution images similar to those recorded from *Plat*_*MR*_ (GSD = 0.7 cm/pixel). In our study, the high-resolution images recorded from the *Plat*_*MR*_ captured a higher variability within the vegetation, probably making the CSM reconstruction more susceptible to slight plant movements and changes in illumination conditions within the canopy. Future studies or applications should consider these factors when planning to use high resolution imagery for 3D surface reconstruction. Otherwise, our study shows that a GSD of (GSD = 2 cm/pixel) may be sufficient for an efficient plant height estimation.

Environmental conditions during data acquisition can also lead to noisy point clouds that hinder PH estimations. These factors could result in lower 3D point accuracy during the orthorectification processing, affecting the point cloud densification step (Tirado et al., 2019). Indeed, our results for HiBAP-I Y17 show evidence that the PHaerial accuracy increased as the wind speed decreased, in contrast with the solar radiation that only slightly influenced the correlations (with no significant differences across GS) in HiBAP-I Y18 (Supplementary Figure S1). Other environmental factors that can potentially have an impact on the PHaerial are shadows (Jin et al., 2017; Brocks and Bareth, 2018), radiometric calibration (Mafanya et al., 2018), brightness levels (López-Granados et al., 2019), and cloudy weather (Niedzielski and Jurecka, 2018).

The UAV/PPK results in our study showed a high agreement with those obtained from CPs and GCPs. This demonstrates that PHaerial using PPK corrections could be an affordable method to increase image georeference accuracy by reducing human interference such as surveying GCPs, importing and manually marking them into the software (Figures 2, 3). As we elaborated in our study, correct calibration of the camera also depends on proper image georeferencing based on the distribution of a sufficient number of GCPs across the field and extensive overlapping between images (Madec et al., 2017; Sanz-Ablanedo et al., 2018). In other studies, RTK/PPK has been used to correct the location of drone mapping imagery and improve the accuracy of GNSS data or geotags as they are captured either during flights (RTK) or after flight (PPK) (Forlani et al., 2018; Padró et al., 2019). However, these previous studies haven’t concluded whether direct georeferencing using RTK/PPK will supersede GCPs to become the standard referencing technique for UAV imagery. The challenges of surveying the GCPs and keeping them in place throughout the life of the crop makes the PPK method cost-effective. It is also time-consuming to check all GCPs on the photogrammetry software to compute the keypoints on the images. To avoid this issue in a practical field situation, we recommend the use of a UAV/PPK system as implemented for *Plat*_*FW*_, since the results were comparable to those orthomosaics georeferenced with GCPs.

Finally, the settings used in the software for orthomosaics and the DSM generation could also impact the accuracy of PH estimation using UAVs. As discussed by Holman et al. (2016), the software settings should be carefully selected and accurately reported for future improvements in UAV-based SfM methods. Our study achieved reliable outputs after testing different combinations of the settings in Pix4D. The optimal settings can be replicated according to Supplementary Table S1. Additionally, we provide a user-friendly script to perform the pixel values extractions using an open-source software (R software) to support future user.

### Accuracy and Phenotypic Variations in UAV-Based Plant Height

The strong correlations and CI observed between PHground, and PHaerial values indicate that this approach can be used for growth rate analysis and wheat selection in a breeding pipeline. The UAV data processing chain used in this study demonstrated itself to be quick, cost-effective, and accurate for the target trait. Moreover, our results showed accuracy levels similar to previous studies (Table 1) for PH estimation at individual GS, with higher correlations at late GS, matching findings of other studies (Hassan et al., 2019a). Nevertheless, it is essential to note that in some studies, the authors report correlations across stages along the growth cycle (Madec et al., 2017; Yue et al., 2017; Yuan et al., 2018; Harkel et al., 2019; Lu et al., 2019), masking the actual assessment power of PHaerial by stretching the response variable (i.e., ground PHs).

Efforts using high-throughput to estimate PH have also achieved reliable results using other platforms in several crops (Harkel et al., 2019; Reynolds et al., 2020). In particular, ground-based light detection and ranging (LiDAR) has been reported to provide more accurate PH estimations than UAV photogrammetry in wheat (Holman et al., 2016; Jimenez-Berni et al., 2018; Deery et al., 2020). However, the implementation of such a platform is limited and can be expensive (Nex and Remondino, 2014; Guo et al., 2018) compared to high-resolution RGB cameras. In addition, cutting-edge technologies in remote sensing have triggered the rapid development of affordable high-performance sensors (i.e., RGB, multispectral and hyperspectral cameras) and UAVs with higher autonomy and payload capacity, increasing the possibilities for field phenotyping applications (Sankaran et al., 2015). Our workflow using UAV-based imagery may be comparatively cheap and more efficient than ground platforms for phenotyping large and multi-location trials, targeting quick data acquisition and reducing computer resources; concepts that are supported by the literature via SfM approaches (Wang et al., 2018; Reynolds et al., 2019).

Despite the high correlations and acceptable RSME of the fitted model, in early stages (i.e., E+40 in Figure 6), the limited range of variation of PH can limit the correlations as demonstrated in HiBAP-I trials (Figure 4), which was also found by Madec et al. (2017). The deviation between PHground and PHaerial obtained in this study corroborates with errors in the literature in wheat PH estimations using UAV platforms (Table 1). In this study, we assessed the *RMSE*_*dev*_ by the agreement between PHground and PHaerial as a measure of accuracy. Studies suggest that bias in crop height estimations by UAV platforms is due to the inability of SfM to reconstruct the top of the canopy accurately (Madec et al., 2017), the influence of neighboring plants (Khanna et al., 2015; Watanabe et al., 2017), and an inaccurate DTM strategy for pixel value extraction (Hu et al., 2018). However, the performance of SfM reconstruction could be improved by increasing the image overlapping (Seifert et al., 2019), and possibly by using better quality camera lenses and shortening flight time to avoid different sky conditions during flight timing. In our study, using fixed-ground targets at different heights proved to be an essential validation step in the current data processing workflow for PH estimations. The very accurate height estimations of the ground targets achieved using *Plat*_*MR*_and *Plat*_*FW*_ (*R*^2^ > 0.95 and RMSE < 4.11 cm; Figure 3), show the real potential of this method, and suggest that differences in plot-level estimations of PH between the PHaerial and PHground may be partly related to inaccuracies in manual scouting over very extensive field trials and wind movement.

In our study, the lack of correlations in HiBAP-II at booting highlighted some issues with PHground that are easily detectable when drawing a transect to compare trend lines (Figure 5). Accurate phenotyping is fundamental for the calibration or validation of novel HTP approaches (Araus and Cairns, 2014), as reported in studies on high-throughput genotyping (Ma et al., 2014). Reynolds et al. (2019) discuss the cost-benefit for phenotyping, showing that UAV-based photogrammetry is relatively affordable when flights operate under favorable conditions, i.e., with no rain, sunny days, and light to moderate wind speed. However, during the flights performed at booting in HiBAP-II Y18, the weather conditions for wind speed and solar radiation were reasonable compared with the other GS in the same trial. The weak correlations in this case could be attributed to heterogeneity within and between plots and canopy architecture issues in detecting the booting during the vegetative stage in experimental wheat plots (Torres and Pietragalla, 2012; Rosyara et al., 2019).

The accuracy of the DTM is paramount for accurate estimations of PH, especially in highly dense canopies such as those observed at A+7 and M (Bendig et al., 2014; Iqbal et al., 2017; Yang et al., 2019). The DTM can be obtained from measurements over the bare soil before the vegetation grows, as performed in this study. Additionally, the DTM can also be generated when vegetation is present by means of point cloud classification (Pix4D, 2018). However, the main challenge of this latter method is that generally at late GS, the bare soil is rarely exposed close to the region of interest during flights to capture in-field pixels. Furthermore, as the detection of bare soil pixels is usually done by image classification methods, this can be affected by rugged relief (Hassan et al., 2019a). Despite these limitations, some authors prefer the estimation of DTM from vegetation DSM, arguing that there are advantages in terms of processing time (Zhang et al., 2018; Hassan et al., 2019a). In our study, we use as a baseline a DTM generated from bare soil images acquired before plant emergence. The advantage of this approach is that it does not rely on image classification algorithms. A drawback of using such DTM is that an extra flight is demanded and more reference points for the SfM algorithm are needed.

### UAV-Based Plant Height as a Reliable Trait for Wheat Phenotyping

The satisfactory correlations (*R*^2^) between PHground and PHaerial observed in this study indicate the applicability of our study’s UAV-based workflow. However, this may not serve all the needs of plant breeders, who often use heritability as a measure of the precision of trials and/or to compute the response to selection (Piepho and Möhring, 2007; Schmidt et al., 2019). Therefore our study also used heritability ($H_{g}^{2}$ and$h_{a}^{2})$ to confirm the UAV-based approach’s ability to infer the predicted genetic values. Additionally, we used the genotypic correlation to compare the similarity between PHground and PHaerial rankings using the predicted values.

We found that the highest $H_{g}^{2}$ values across GS for PHaerial may indicate more reliable phenotyping measurements. In this case, the selection ranking of the best genotypes could be done using PHaerial assessments. This finding was also confirmed by the Spearman rank correlation (Figure 7). Therefore, our workflow for phenotyping PH combined with reliable $H_{g}^{2}$ can be an affordable and efficient method to offer breeders more accurate genotype selection criteria. Other studies have also supported a link between higher heritability (or repeatability, in some cases) and PH in later GS (Hassan et al., 2019a; Deery et al., 2020). However, some issues may appear in the temporal image when the target traits depend on the geometric structure, as described by Madec et al. (2017), who observed poor $H_{g}^{2}$ at the end of the growth cycle due to plant lodging. These circumstances were not evident in our study.

Medium to low levels of $h_{a}^{2}$ observed in the GS suggest a meaningful environmental influence, indicating that G × E interactions affect PH predictions (Figure 8). These results were supported by the random effect significance (Wilks, 1938) of G × E interaction at most of the GS analyzed, except in E+40 for *Plat*_*MR*_ and PHground (data not shown). The non-significance, in these cases, can be attributed in part to the limited range of variability for PH. Furthermore, lower $h_{a}^{2}$ are expected when compared with $H_{g}^{2}$ due to pedigree information, in which the $h_{a}^{2}$uses the proportion of genetic variation due to additive genetic effects only (Piepho and Möhring, 2007). The results confirmed that PH is a critical trait responsive by G × E interaction, as expected in quantitative traits (Tian et al., 2017; Tshikunde et al., 2019). The high correlations between predicted values for PHaerial and PHground across and within GS indicate that each platform measured similar underlying genetic traits. This means PHaerial can reliably predict genotypic values and rank genotypes as reliably as PHground.

## Conclusion

The present study implemented and validated an efficient and scalable approach to acquire PH measurements under extensive wheat breeding trials at different growth stages. The remote sensing techniques applied in this study allowed the estimation of PH using high-resolution RGB imagery recorded from two UAV platforms and processed through a semi-automatic pipeline. The results for all trials in two growing cycles prove that the study workflow was able to estimate PH from UAV platforms comparable in accuracy to those measured by ground-truth notes. Our findings also indicate that using PHaerial for genotype selection could be a cost-effective way to predict PH values using temporal data from drone imagery taken in multiple environments, mainly in late GS. Due to the reliable results achieved by *Plat*_*FW*_ to compute PH, it is reasonable to conclude that a lower density point cloud does not confer PH noise or underestimation in comparison to *Plat*_*MR*_. The accuracy was responsive to image quality (i.e., GSD, weather conditions, etc.) and the settings in the processing steps of the surface model generation. A proper georeferencing of the orthomosaic is an essential step for data extraction, and the UAV-PPK approach was demonstrated to be a suitable method to replace laborious conventional methods using GCPs.

As evidenced by wheat PH studies in Table 1, different pixel extraction approaches can be made by choosing different thresholds for capturing the genotype variability within and among experimental plots. In this study, the reliably results obtained using PH estimations at multiple GSs and environments was also endorsed by the authors in Table 1. Finally, this study demonstrates that it is feasible to process high-volume field-based phenotypic data using UAV-based imagery.

## Data Availability Statement

The raw data supporting the conclusions of this article will be made available by the authors, without undue reservation.

## Author Contributions

FR conceived and designed the study and supervised the project. LV, LG-P, and IT collected and analyzed UAS data as supervised by FP and FR. LV performed the imagery quality analysis and conducted the literature survey. LV and LG-P performed image analysis, analyzed the data, and developed the statistical code of the study. LV, FP, LG-P, IT, and FR conducted the Remote Sensing component of the study. LV, FP, and FR drafted the manuscript. AB, MR, BG, and GM provided critical insights into the manuscript writing. All authors listed have made a substantial, direct, and intellectual contribution to writing and revision of the manuscript and approved it for publication.

## Conflict of Interest

GM was not employed by company KWS Momont Recherche during the execution of this research. The remaining authors declare that the research was conducted in the absence of any commercial or financial relationships that could be construed as a potential conflict of interest.
