# Supplementary material for: High Throughput Field Phenotyping for Plant Height Using UAV-Based RGB Imagery in Wheat Breeding Lines: Feasibility and Validation
Source: Front Plant Sci. 2021 Feb 16;12:591587. doi: 10.3389/fpls.2021.591587 (PMC7921806; doi:10.3389/fpls.2021.591587)
Supplement: Supplementary file 1 [file Data_Sheet_1.docx]

Supplementary Material

**Supplementary Figure**

| 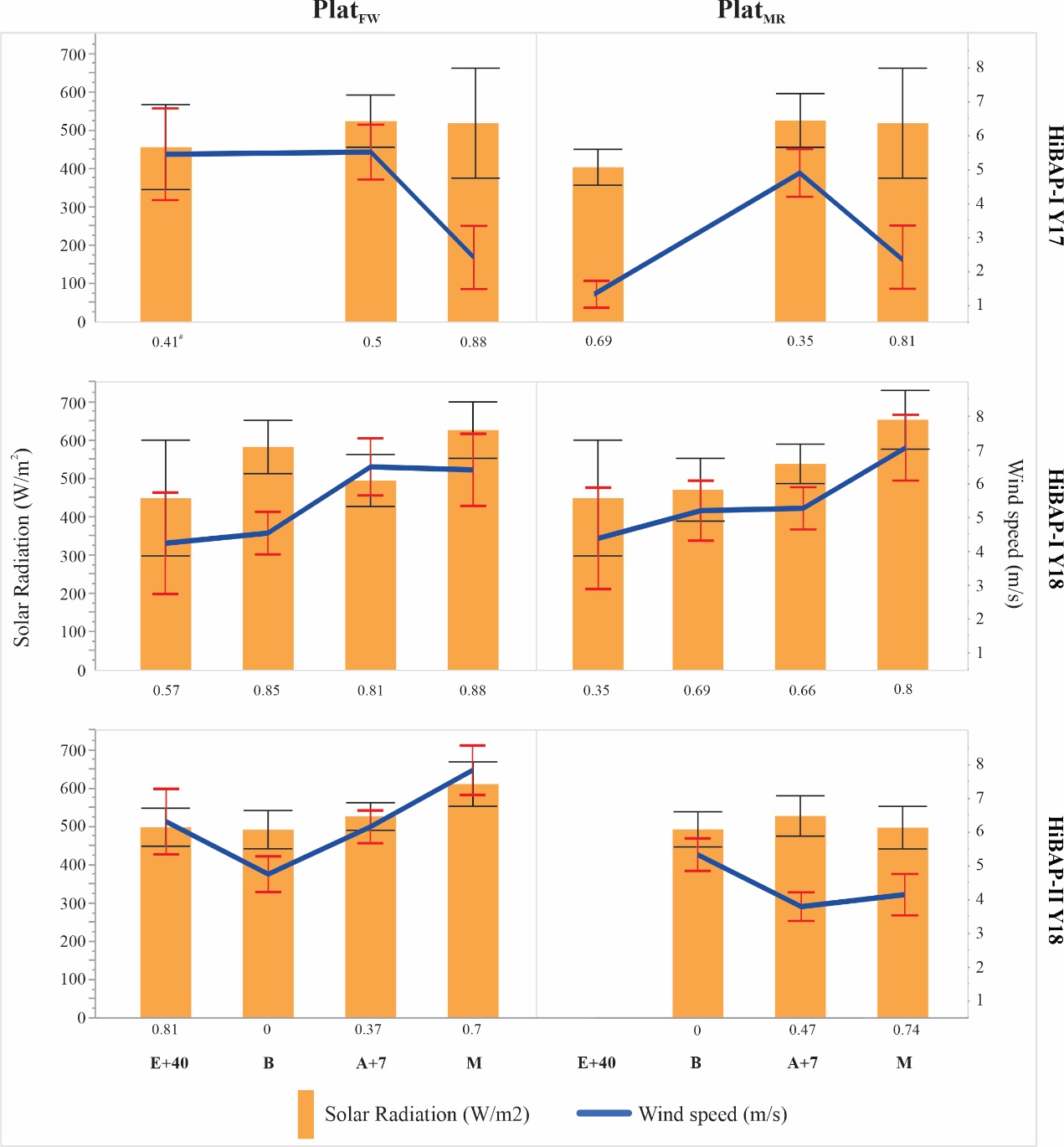 |
| --- |
| Supplementary Figure S1. Average of flight environment conditions for two breeding cycles around the time of flight campaign at 40 days after emergence (E+40), booting (B), seven days after anthesis (A+7), and maturity (M). The black and red cross lines represent, respectively, the 95% confidence interval computed for solar radiation and wind speed.  **^#^**R-squared (*R^2^*) value between PHs ground-truth measurements (PHground) and UAV-based estimations (PHaerial). |

**Supplementary Table**

Supplementary Table S1. Pix4D processing parameters for generating the Point Cloud Densification and final DSMs details for fixed-wind (${Plat}_{FW})$ and multi-rotor (${Plat}_{MR}$) platforms.

|  | **HiBAP-I Y17** | |  | **HiBAP-II Y18** | |  | **HiBAP-I Y18** | |
| --- | --- | --- | --- | --- | --- | --- | --- | --- |
|  | ${Plat}_{FW}$ | ${Plat}_{MR}$ |  | ${Plat}_{FW}$ | ${Plat}_{MR}$ |  | ${Plat}_{FW}$ | ${Plat}_{MR}$ |
| Keypoints image scale | 1/2 | 1/2 |  | 1/2 | 1/2 |  | 1/2 | 1/2 |
| Image Scale Quality of Point Cloud Densification | 1/2 | 1/4 |  | 1/2 | 1/4 |  | 1/2 | 1/4 |
| Average Number of 3D Densified Points^#^ | 3385701 | 7024707 |  | 5716299 | 4155111 |  | 1991084 | 6565985 |
| Average Density of point cloud (per m^3^)^$^ | 569.73 | 3950.22 |  | 1566.28 | 2697.50 |  | 1739.54 | 2423.69 |

^#^and ^$^are the total number of 3D densified points obtained, and the average number of 3D densified points obtained for the project per cubic meter, respectively, across all projects performed in this study.
